# Autobiography of the Insane

**Published:** 1856-04-01

**Authors:** 


(*To be continued.*)

---

### Art. IV.—AUTOBIOGRAPHY OF THE INSANE.

(*Continued from page* 82.)

I am dead. Like Seneca, I have been bled to death by my persecutors, who each had a cupful of my blood. I well remember that I have suffered death with resignation, and praying to God that he would forgive me my manifold sins. My voice has been heard; but I am not yet worthy of being numbered among the *Elus.* My good and bad actions during my lifetime are carefully weighed by our Supreme Judge; the latter are too numerous, but my repentance at the hour of death is taken into consideration. According to my belief, as a Roman Catholic, I am doomed to pass a certain lapse of time in Purgatory. My murderers have already been overtaken and struck by the justice of men. They did not repent; they are damned for eternity.

In atonement for my sins on earth, and before I may obtain the kingdom of Heaven, I must be put to the test, and tempted by the Infernal Powers for several hours every night. I am, therefore, carried into the dominions of Satan, who endeavours, by a display of magic operations, to show me that his *puissance* is too great to be resisted successfully, and that sooner or later I

shall give way. He also tries to persuade me that he can make me more happy than I am in purgatory. He points to a number of his subjects, among whom I recognise my persecutors, who seem to enjoy their present position.

For the first time, I feel an invincible courage within myself. I firmly answer that I despise him, his threats, and his promises, and that, with my God's assistance, I fear nothing, and may defy all the monsters in his dark kingdom. The room then assumes a more gloomy appearance; it is vaulted like a cellar; a sulphuric smoke comes out of the fire-place, so thick as to conceal many objects from my sight, and to stifle me. The walls are covered over with grimacing, horrid monsters, at all of which I now laugh fearlessly, saying that this is nothing compared with what I saw many a time when I attended theatres. Now and then, if I perceive that the attacks made against me are too powerful, I am, as it were, inspired to have recourse to prayer. I therefore repeat uninterruptedly, aloud, and in any language I know, our Lord's Prayer, which I had nearly forgotten, together with *Glory be, &c.*; or I sign myself. I remark that no one of the devil's attendants, or even himself, dare to touch me while I am praying. On the contrary, they seem to suffer a great deal inwardly, and slink away, in uttering curses; but they return to the charge as soon as I leave off praying. After a long, a very long struggle, I grow weaker and weaker; I can hardly speak for want of a drop of water, which I would not, however, accept from unholy hands. I am smothered; perspiration flows down my cheeks; my strength is exhausted; the evil spirits profit by my prostration; I feel crawling about me and on me repulsive reptiles or animals, such as serpents, toads, frogs, rats, mice, &c. There are myriads of them. Their size is so large that I must see them through a microscopic glass. Here my good angel comes to my deliverance. I am carried back to purgatory. Now I fall asleep.

I have slept until eight o'clock—three or four hours, I should think. My head is clearer; I am not so restless; the noise in my ears is lighter. The two other patients tell me that I had a very bad night. My eyes were rolling in their sockets, like those of a madman. I was very noisy. I seemed to fight for a long time. I spoke sometimes in Latin, sometimes in English, but mostly in an unknown language. The night-nurse wanted to make me drink, but I could not, even with the assistance of the cook (man). It was fortunate that I could not stir.

The doctor comes in, and finds that my pulse is less agitated. Notwithstanding the nurse's report about my excitement of last night, he orders that I should be free in my movements. The strait-waistcoat and other *courroies* are taken off. No visions until night, when I fancy that Satan himself is lying by my side. I also imagine that my persecutors have resumed their places in the other beds. They say that it is a shame. I am sleeping with the devil. They see his long flat feet hanging out of the bed, and from which I try in vain to disentangle mine. In my opinion, Satan has taken the shape of a wolf. His head is remarkable by two short horns. The whole of the body, with the exception of the two feet, which are as cold as ice, is covered with long yellow hairs, emitting a most nauseous smell. He again speaks to me in a threatening manner. I do not listen to him. My only answer is, that I no longer fear his power, because God is my protector. Then I commence to pray, sometimes in a low voice, sometimes aloud, but always composedly, as if I felt quite safe. I still hear the once dreaded voices; but reason seems to have returned—she tells me not to trust sounds.

This was, Monsieur le Docteur, the third night I had visions since my admission into the infirmary; it was also the last one. From that time the visions completely vanished. It is true that I was still very far from being restored to health. My sight was greatly impaired for some more days. My appetite did not return all at once, but by degrees, and accompanied by a good sound sleep. I here must acknowledge that nothing was spared by the medical gentlemen which was likely to accelerate my complete *guérison.* They told me I had been very ill; and indeed I think they entertained very little hope of my recovery. I left the infirmary, when I asked for my dismissal towards the 20th of February.

On my return to town, I felt much more inclined to live as a Christian. I could not help believing that all the events, either real or imaginary, had taken place through God's will for my conversion. My first care was to consult the priest, and to take his advice about marrying the girl, notwithstanding my gloomy recollections concerning herself and her family. The reverend gentleman owned that the connection was altogether unsuitable, that it was a great pity, &c. But I had a great sin to expiate. Marriage had become a necessity.

We were therefore married, although I had forgotten nothing. I first wanted to quiet my conscience, and was very much like a man who clings to any plank of safety, however rotten, to avoid drowning. In consequence of my new connection, now openly known, I lowered my usual charge, thus hoping that many pupils would avail themselves of it to learn French. My business was resumed without any loss of time. I abstained from any strong drink, and should have most likely been enabled to maintain my little family, had not my wife been badly advised by her friends, who did not dare to come to my house, but whom she visited.

For reasons I cannot explain to myself, they, without any means of a livelihood, had taken up lodgings in Derry. I found her several times, when she returned from those visits, in a state bordering on intoxication. I then saw that the fruit of my labours was again going the wrong way. I got discouraged—disgusted with life. I drank again, lost my appetite, experienced new fits of faintness (no visions), accompanied by diarrhœa, and finally by want of sleep. My little money being gone, the pawn-office was resorted to; my watch and clothes were engaged, piece after piece, until there was nothing left. Then I saw that my only resource was to risk my return to France, after gathering up two or three pounds remaining due to me for tuition. My books, together with some furniture, were left to my wife, who, it was agreed, would try to live with her family until I should be able to get a situation, after my recovery, either in France or in England. She did not look much annoyed at my departure; but it is not the less my intention to discharge my duty as a husband as soon as Providence is pleased to give me the means. I would now work for her and the child much more than for myself. May this also be a lesson to her!

Having described to you, Monsieur le Docteur, what I call the first period of my disease, I will now give you an account of the second. When I left Derry, I had kept my room for two or three weeks, being unable to go on with my lessons, though the soundness of my mind was not once impaired again there, but from mere exhaustion. I resume my diary:—

Left Ireland on the 26th of June, with some cakes and a little bottle of whisky. For saving expenses, took the steerage: could not eat; drank the whisky; no sleep during the passage; very feverish; suffering much from diarrhœa. Arrived at Liverpool, 27th; no food, but one or two pints of porter. I feel very, very weak. For fear of being taken sick on my journey, and placed in the impossibility of proceeding, I take the mail-train, in order to get home sooner, there I have to pay 4*s.* 6*d.* more than I expected. In the carriage I endure great sufferings from vomitings. My stomach being empty, I expectorate nothing but bile. I can hardly sit up. No more sleep than on the preceding night. On the 28th, arrived in London, with about 10*s.* in my pocket. I am exceedingly depressed in mind, and wearied all over my limbs. I want to apply, Portman Square, at a relation's temporary residence. I inquire of many persons about my way. Their informations are very conflicting. At last I reach my destination, after a walk of more than three hours.

The people of the house answer me, that my cousin returned to Paris three weeks ago.

This sad announcement adds, if possible, to my despondency. There is my last hope gone, as to the possibility of getting home without a stoppage on my way. I can, however, through great economy in my expenses of the day, manage to save *eight* shillings for my passage to-morrow, on board the Boulogne steamboat. Once in Boulogne, I shall at least be in France, and, as I carry about me my passport, my degree of A.B., with a great number of excellent testimonials, I may hope to interest the authorities in my favour, and to obtain from them the means of proceeding on my journey.

I continue my walk for many hours, now and then stepping into a public-house to take a glass of ale, or ginger-beer, when I feel too thirsty; but I do not taste any more substantial nourishment. It seems as if my stomach could not digest it. Though broken down with fatigue and hardly able to stand up, I very seldom stop for a few minutes' rest. I feel that stopping is still worse than walking; because the absence of objects constantly renewing deprives my mind of diversion, and makes it a more easy prey to thoughts of despair. I therefore go on, unconscious and unmindful of the direction I may take. In a narrow and dark-looking passage through which I wander, a few French words fall on my ears; I turn round and find that they come from a man, in a small stall, who sells cheap ices at one penny each. Being anxious to get a modest bed-room for the night, and in the hope that the man can give me some information about it, I enter the stall and ask for an ice; then I beg the permission of sitting on a chair; for, said I, I have been walking a great deal and feel very tired. The *ice-dealer* gives me a chair; he then inquires of me if I am a foreigner; on my affirmative answer, he says that he is a native of Switzerland, but knows France very well. He was there for several years. I perceive that he does not speak English, or at least pretends not to know it. I see in the stall two grown-up boys employed as assistants, and with whom the Swiss converses in bad Italian. A great many customers, mostly of the poor classes and of little prepossessing appearance, come in and ask for an ice. Some appear to be acquainted with the man, although he has just told me that he commenced business this very morning. No suspicions however strike my mind. I frankly confess my distressing state; I should be very much obliged by his taking me to a lodging-house where I may obtain a bed for the night; I want to take the Boulogne steamer to-morrow, and I have just enough for a bed, in a very modest lodging-house. The Swiss, after much musing, takes me to a place where, he said, I shall be well.

Despite his assertions, however, I have no sooner set my foot in the house than I wish I had never come. This is a most miserable-looking place, situated in a neighbourhood which can have no claim to respectability, from the number of rags and repulsive individuals I have met on my way. I am conducted, through a dark alley, up to a kitchen on the first story. The landlord and landlady to whom I am handed by the Swiss, in a few Italian words, are not likely to restore me to confidence. The former is a tall, lean fellow, about fifty years, wearing moustaches, and smoking a clay-pipe by the fire-place. Were I in France, I would take him for a *coupe-jarret.* His wife is an old woman whose face has been greatly injured by the small-pox and the loss of one eye. I find her very ugly. There are two young women in the kitchen engaged about I do not recollect what. They certainly have bold looks. Several *orgues de barbarie* and *grosses caisses* let me guess the kind of companions I shall have for the night, if I have nothing worse.

The old woman invites me to take a cup of tea. I decline accepting of anything, and express my desire of retiring to rest immediately, for I cannot sit up any longer, from weariness. She leads me through a very steep and dirty staircase to a room containing three beds. One of them I may have. Before leaving, she wants me to pay in advance the usual charge,—sixpence. When I find myself alone, I take a survey of the place. One table, the three beds, and a few common chairs, make up the whole furniture. I again observe a big drum on the floor, which affords me another proof that showmen as well as strolling singers are the customary lodgers of the house. No sinister suspicions, however, throw my mind into distrust and fear. I address a sincere prayer to God; I think, when in bed, of those I have left behind. I cannot help shedding tears; but I hope in better days. So far as I can judge, it may be six o'clock. I have therefore been walking many miles since six in the morning. Sleep soon overcomes me. I have no evil dreams; but a noise in the room puts an end to my rest. I awake abruptly, and look about to see what the matter is. The night has come. I see the old woman holding a candle. She is with a man and a woman, whom she leaves an instant after. My two companions take one of the vacant beds. The woman looks very much like one of the two females I saw; but the man is not at all the same as the tea-dealer, although the landlady told me, when I was conducted to this room, that he sleeps there every night. Both begin to talk in a low voice. From their conversation I perceive that they believe I am asleep. Imagination again arouses my terrors. I fancy that they speak sometimes in French, sometimes in English. I wonder how they have come to a knowledge of my language, especially the woman, who expresses herself with great correctness and a truly good accent. Then I imagine that she may be one of those Frenchwomen, so numerous in London, whose existence is derived from debauchery or theft. I think that this one, after acting her part on the first stage, has now fallen into the second. In short, I firmly believe that she is connected with a gang of robbers. They, said I, intend to get rid of me, in order to obtain possession of my few shillings. I suppose there is a weapon, such as a dagger or a sword, concealed under their bolster. They seem to encourage each other in their murderous design. "*How much has he got?*" asks the man. "*Only eight or nine shillings,*" answers the woman. "*It is a poor job; but we must get it.*" Moreover, there are my clothes, with a small parcel in which they will perhaps find something better. After much arguing, they at last agree to wait for the arrival of other friends who are to sleep in the third bed.

Such is now the state of my mind, that I would swear my life is actually in danger. I pretend to awake suddenly; I don't appear to have overheard any part of their conversation. I cough, and often complain of weariness. I keep myself in constant fidgeting, as if it were quite impossible for me to sleep any more. I thus hope to deter them from their criminal intentions, and, indeed, I hear them uttering curses and imprecations because I do not sleep again.

At a late hour in the night, there is a great noise below. Many people, males and females, are uttering coarse jokes, or singing and disputing. Decidedly, this is not a respectable house. I feel more afraid than ever. Two men come upstairs with a girl. They talk such English that I cannot understand. I suppose it is *argot* (slang). The girl stops at the door of our room, and shows her two companions into it. Owing to darkness, the countenances of the new comers are not to be distinguished. What I can perceive is, that one of them is very tall, and the other of middle size. They enter into conversation with the man and woman, but they use a language unknown to me. This fact increases my fears. Should I be sleepy, I feel that I must not sleep, because I am not in a safe place for rest. After a long talk has been going on in a low whisper between them all, except myself, they bid one another good night; but I observe that they remain wide awake. I move about to show them that I am not asleep either. They appear to be much disappointed, and utter frightful oaths. At times, there is a noise from the story above, as if produced by the fall of a piece of furniture, or by the rolling of a bowl. Voices from outside the door address my companions, urging them to have done, because the night is far advanced. I remark that these pretend to snore, but never all together. There is but one snoring at once, and each differently from the others. They alternate. I moreover remark that the noise from outside takes place when there is no snoring at all. Again, the said snoring ceases when they imagine, from my immobility, that I am asleep. I come to the conclusion that they have agreed to lay hands on me during my sleep. They will smother me with the bolster, and, in case of a noise on my part, will stab me in the bed. I hear that it will not require much time for them to dig up a grave in the yard.

I give myself up as lost. I pray that the day may come. But the night is still far from being at its end. A clock from a neighbouring church strikes every hour. It has just struck one. It seems as if it were a signal. The silence of the night is suddenly interrupted. People in the street—men and women—raise their voices to a stunning pitch. They swear, sing, laugh, and dance. They shout out that it is quite time that *the cat should be bled.* Then a mourning-tune (*un air de deuil*) is sent forth from an *orgue de barbarie,* and brings to my bewildered mind a most sinister recollection—the horrible *assassinat de Fualdès* which occurred in the South of France some thirty years ago, and during the perpetration of which, an accomplice to the murdress was engaged playing airs on an *orgue de barbarie,* in order to keep the attention of the passers-by from the *sanglant* theatre. Though many years have passed since I read of it, I can now remember the most insignificant particular. My memory serves me but too well, for such recollections make me the more uneasy and incapable of reasoning.

As if the boisterous scene in the street wanted any accompaniment, I hear, on all sides, howlings, barkings, whistlings. It seems that all places around contain a swarm of ferocious animals, who are well aware of what is to be done with me. Rude, angry voices often address the individuals in the room to make haste; but every time the latter, however, reluctantly give the same invariable answer,—*There's no go.* At intervals, too, the rattling of a cart, like a tumbrel, passing and repassing at a furious speed over rough, hard stones, contributes its quota to that infernal concert, which, in my opinion, is made to drown any cries on my part. I am first surprised that there is no night-watch to put a stop to the disturbance. But I soon observe that whenever the approach of heavy footsteps is heard, the gang receive information of it, and the noise is immediately hushed, to be continued as soon as the sounds of the said footsteps have died away.

My mind is thus tortured until daybreak. A faint hope penetrates into my heart. I cast stealthy looks about me. My companions do not sleep, for they are very restless. I suppose they have not yet given up their bad designs. I then examine carefully if there is no means of escape. Unfortunately, my *examen* confirms the worst suspicions in reference to the house. On my right, the window is secured by iron bars, and overlooks a small, dirty yard, surrounded by nothing but walls. My eyes turn to the other window, which is opposite a red tile roof, and so close to it, that I imagine I might jump out on that roof, were not the window exactly situated between the two beds occupied by the other lodgers.

Being therefore convinced that all hope of escaping through the windows is to be abandoned as chimeric, I resolve to defend myself to the best of my power against the attack I expect every minute. There are in the small parcel I brought with me two razors and a penknife, in the pocket of my trousers. I take out one of the razors and the penknife, which I open in silence, and which I place beside me on the bed. My companions have perceived these preparations. They seem to laugh in disdain at my means of defence. I think they say that the struggle will not be a long one. The idea of a longer weapon being in their possession, such as a dagger or a sword, again recurs to my mind. I then venture to speak. In a most trembling and scarcely audible voice, I say that I know their intentions against me, &c. I am determined to sell my life dearly. Perceiving that my words do not appear to produce any effect on my audience, I appeal to their humanity. I entreat them not to steep their hands in my blood, especially for such a trifling sum as *eight* or *nine* shillings. I am to return to my country this very morning. If they allow me to go, I promise to leave London without making any disclosures about them and this house. Let them take my money, if they like; I shall not complain.

I go on for some time in the same strain; and at last, seeing that all my supplications seem to remain unsuccessful, and that the men will not alter their minds, I beg of them permission to grant me only a few minutes as a favour. No answer. I hastily slip out of my bed, fall on my knees by the bed-side, and say a short prayer in a low voice. I feel a great deal more composed. There is now so much resignation in me, that I no longer fear death. I tell my companions that I am ready; they this time say that they wish me no harm. Though I do not believe in their friendly protestations, my terrors are gone. Let them strike me while asleep. This reflection does not prevent my taking two or three hours' rest, until seven o'clock strike by the church clock. The other men are still in bed; one of them gets up at the same time as I do, because, says he, the doors below are not open. He leads me down the steep and narrow staircase. I find myself in the kitchen I saw yesterday. My guide is the tall man I remarked last night; he says he is the landlord's son. He takes me to the street door, and accedes to my request, when I express a desire to be put in my right way to London-bridge. He therefore accompanies me for some minutes, and leaves me in a wide street, saying that I have only to go straight on. I forgot to mention that he handed me two cards, to recommend the house to my friends, should any of them come to London. Those cards I took, but without any intention of ever using them as I was directed. They have been taken from me at the house where I was before my being brought here. The landlord's name is Cassanello (an Italian).

I have been told by my guide that London-bridge is about a good mile off, and that the shortest way for me is to keep straight on. I therefore forget my state of exhaustion, and walk at a brisk pace in order to be in time for the steamboat which is to sail at nine o'clock. I have already proceeded for not less than one hour, taking great care to follow the same endless street. There is, however, no London-bridge within sight yet. I venture to ask a policeman about it. He informs me that I am *three miles* at least from my destination, and points to another direction as the right one.

On this day, Sunday, 29th of June, disappointments succeed disappointments. It seems as if London-bridge were moving and retiring before me as I advance towards it. Despite repeated inquiries, I think I should never have reached it, had I not at last and in despair given a little boy one sixpenny-piece to take me there. It was twelve o'clock when I arrived; the steamer was gone, and with her my last hope of leaving London on that day.

I see everywhere people going to their places of worship. An interior voice tells me that it would be right on my part to do the same; for I stand in extreme need of our Lord's assistance. But on casting a look on myself, I feel ashamed of my wretched appearance, and content myself with praying to God that He may deign not to abandon me. I go on at random until the divine service is over; then I enter a public-house for the purpose of writing to my family, and apprising them of my being detained in London by illness, and unable, for want of pecuniary means, to proceed on my journey. When I have done, I recommence my wandering *marche* without interruption, without food, until night. I have been all day exposed to a scorching sun: I feel quite worn-out; but I continue walking, like a machine, an automaton, without caring about any direction whatever. It is my intention to apply for lodgings to any policeofficer I may meet on my way, when the streets are getting desert. I thus hope to obtain a bed in a respectable house.

At about ten o'clock, I find myself in a wide thoroughfare, where I see thousands of promenaders moving along the footpaths. From distance to distance, the landlords of several public-houses have placed rows of chairs and forms, with tables, in the street. There sit many, many people, drinking beer and eating cakes. I am very thirsty, but I would not take any beer, because I am sure it does no good. I buy a cake, and draw a little water out of a pump.

I then resume my walk for one hour perhaps; I perceive that the streets are not so thickly filled with people now, that it will soon be time for me to think of some accommodation for the night. Were it not that my step is more unsteady, my voice more trembling, my sight weaker, and my hearing subject to a constant humming, I feel nothing which may induce me to believe that I am worse than I was this morning.

Presently, and all of a sudden, the real scene changes, so far as people are concerned. This is the same street, indeed, with the same buildings; but the promenaders, the women especially, are no longer strangers to me: they have assumed forms with which I am acquainted; I shudder on recognising in two females the faces of my wife and her sister passing and repassing beside me; they are laughing a diabolical laughter; they cry out that I am *mad—yes, mad, and this time mad beyond recovery.* I shall die the death of a brute; I shall be damned for eternity.

There is just enough *présence d'esprit* left in me to think that I am again the sport of a delirious imagination, and that I am destined to suffer under new trials. Notwithstanding the unceasing threats I distinctly hear about me, I wont believe, but at the same time I cannot help being more and more excited, and in spite of myself I answer those menaces as if they were real. It is time to apply to a policeman. After some minutes' walk, during which I get no relief, I find one whom I beg to conduct me to a decent lodging-house, in which I may find a bed for the night. I am a foreigner, quite a stranger in London; arrived yesterday, but would not like to return to the same house I slept in last night, because I think it is a bad one. I am ill, very tired, &c. The officer kindly takes me to a place where he is known. The people of the house, perceiving that I am unwell, desire me to take something before retiring to rest. I decline, and only drink a glass of ginger-beer. As soon as I am in bed I feel very much oppressed. I can hardly breathe. My eyes and mouth send forth sparks of fire. A stormy, hissing wind rages about my ears. All my body is in such a state of perspiration that I put off my shirt. I fancy that a demon is on me, trying to smother me by pressing on my throat. I struggle with all my might, and pray repeatedly. My prayers drive Satan from me; but he is not far hence. I still see his hideous face in the room. The latter part of the night passes away in visions of a new kind. My memory has acquired a wonderful power of recollection. I see, in a succession of *tableaux*, as I should in a panorama, the faithful reproduction of what I have done wrong during my life. Many sinful deeds, never remembered before, and which I believed to be for ever buried in oblivion, now spring up one after the other, and defile before my eyes.

The day has long made its appearance, when I am able to snatch a little rest. At breakfast-time I am still in bed. The landlady has been informed, by two young men who slept in my room, that I was very restless, without, however, being noisy at all. She sends up to me a cup of tea and some toast. I take the tea, with very little bread. I cannot eat. I bought last night half a pound of meat, which remains untouched. When I have got up, I stop for some time in the parlour below-stairs with the landlady, to whom I sincerely confess my penury, and the reasons which compel me to tarry in London until I have received an answer from home. She happens to be a kind-hearted woman, and sympathizes with my sorrows. She accepts the money due for the bed, but refuses to receive anything for tea. I then tell her that, if she has no objection to it, I shall sleep in her house again, a proposal to which she readily consents. I take leave of her, with the intention of taking a short walk, and, in order to get rid of any incumbrance, I entrust her with the care of a small parcel, containing, among other things, my passport, my degree of A.B., and a number of testimonials. Although I have avoided strolling too far away from the place, I vainly endeavour to find it again. That the house is close to a railroad, and I was able to see the trains from my bed, is all I can say; for I have forgotten to ask the landlady for her name and the name of the street. At last I discover a railway which is quite, in its appearance, like the one I am looking for. Indeed, the aspect of the adjoining streets, cut, as it were, into two halves, makes me almost sure that I have come to the end of my anxious rambles. Unfortunately, appearances were never more deceiving. I walk over and over again through some twenty streets in the vicinity of the railroad, all to no purpose. I give up, for fear of being looked upon by the people as a suspicious character. I have thus been on foot for at least five or six hours, being sustained by nothing but ginger-beer, the only sort of drink I made a vow last night that I should taste again.

In the hope that an application to the police may lead to the discovery of my papers, I hurry on to the nearest station, where

I state the case to the best of my abilities; for I have very little strength even to speak. After hearing my statement, the chief officer tells me that it is very unfortunate, he can do nothing unless I let him know at least the name of the street where I met the policeman who took me to the lodging-house. I venture to express my opinion that it would be easy to find out the said policeman, by inquiring at all stations, which of the police conducted last night, about 11 o'clock, a Frenchman to a lodging-house; but all my reasons are not listened to. I therefore submit to try if I can find the street again. The officer tells me that I must come back as soon as it has been found, and assures me that he will spare nothing to have my parcel restored to me. I leave the police station, not at all despairing, in my ignorance, to be able, by dint of turnings and windings about the streets, to find at last the one I am instructed to look for, and of which I suppose I have kept a vivid recollection.

I shall not weary you, Monsieur le Docteur, with a detailed narrative of my new perambulations; I shall only beg to say that, on that day, I did not even so much as sit down for more than twelve hours. I had no kind of food whatever; thirst alone compelled me to stand from time to time at a ginger-beer shop, *en plein air*, where I had a glass of the refreshing drink, and then on I went. I could not stop; it seemed to me as if somebody were again pricking me from behind, or whispering into my ears: *Walk on, walk on.* The objects grew confused. I heard imaginary conversations held in French. They related to me and my insanity. At times the prickings became so painful as to make me shed tears, and it was with the greatest effort that I could help uttering cries. Towards evening I was prompted, I cannot say by what invisible force, to go and give an answer at the police-station as to the issue of my errand. The difficulty was to get to it. It was very likely a good distance away. Frequent were my applications to policemen on duty in the streets, but either I gave them a wrong name, or they did not know the place. The fact is that I never obtained the information I wanted. In fine, and, *en désespoir de cause*, I called at the first station-house on my way, and asked to be taken, if possible, to Finchbury station, (so far as I can remember), where I desired to speak to the chief officer. They kept me waiting for a good while there, and it was dark when I was requested to follow a policeman who, they told me, was going to my destination.

Now, Monsieur le Docteur, I will relate at some length to you the strange events, partly real, partly imaginary, that took place on the night of the 30th of June, from the moment when I left the station-house to accompany the policeman. I resume.

This officer looks angry with me, as if I were a malefactor. I ask him if I have done anything wrong; he answers, *Nothing that I know of.* We have not proceeded many yards out when two ill-looking men come up and walk by my side. Their language is most abusive; they make threatening gestures at me. They say they are going to the station along with me, and there swear before the magistrate that I created a disturbance at their house. I call the policeman to witness that the accusation is quite false: I entreat him, with tears in my eyes, to disbelieve such a wicked report. The men I now take for two of those who slept in my room on Saturday night. They must be bad characters, said I, for they wanted to lay hands on me. The officer does not pay much attention to my supplications; on the contrary, he seems to be on very good terms with my accusers. He soon leaves me in a street, and, on going away, says that we shall meet again at the station, which is now within a few minutes' walk. I have, says he, only to go straight on. The two men are still by my side: they still abuse me; but, notwithstanding what they have just declared, about their intention of having me brought before the magistrate, they also leave me, and proceed on their way at a quicker pace. To my great dismay, I hear them crying aloud—*Here is the madman coming. . . . Here is the madman.* This appears to be *un mot d'ordre* for every one. The two men are certainly new enemies. They try to set up all London against me. Indeed, everybody is standing at his door, laughing at the madman; some speaking with compassion, others asserting that he ought to be locked up for the safety of all.

The unavoidable cry is repeated from distance to distance, as if to invite the people who are in doors to make haste and look out, for there is the madman. I cannot understand how people may be so easily imposed upon by a set of slanderers, and thus rise up against one who does not remember having done any harm. I feel that resistance on my part would be great folly: my only resource is to suffer with new resignation. I, therefore, thinking it useless, throw the walking-stick which I carry over a wall I pass by.

I now go on in a slow quiet pace, with my hands in my pockets. I am entirely composed. Though I would swear to the reality of whatever I hear about me, there is in me an invisible adviser who commands me to bear up in silence against any kind of abuse. Sometimes, however, I cannot help exclaiming: *Je vous reconnais bien là, M. Diavolo; encore un de vos tours contre moi; mais je ne vous crains pas; je vous défie; car je suis sûr que le bon Dieu est pour moi,*—and many like sentences. Once, thirst obliges me to enter a tavern for a glass of ginger-beer. There are three men sitting on a bench in the bar-room; I imagine they speak of me, for I have caught the word *madman.* I complain of their behaviour towards a helpless foreigner, who is only guilty of being poor. They politely answer, that I am under mistake. I am not at all the subject of their conversation. I then apologize for my blunder, and walk away with the conviction that every one has been roused against me. A little further on, I feel inclined to buy a penny loaf; but it seems as if all the bakers' shops were now closing on purpose, and that no one will sell me the food I am in need of. This universal bad feeling I ascribe to Satan's power; but I have full confidence in God,—I pray on fervently, being assured that I shall not be abandoned. How long did my walk last, through hundreds of streets, it is difficult to say exactly. Most of the shops had already been shut for a long time; the thoroughfares are no longer crowded with promenaders. It is very late. How is it that I am neither weary, nor cold, nor hungry? To these questions I know of no other answer than that I am under the care of Divine Providence.

I meet many persons whom I take for acquaintances of mine. They have come to be present on what I call my *Passion.* There is a master whom I knew at Foyle College. He passes by without speaking. There is my brother-in-law, whom I have just passed. I know him well. He has a brown over-coat on, and smokes a cigar. There he is again. He wont leave me; he says he has come to have done with me at last. I presently hear his voice exciting every one to throw me into the river. I defy all in a loud tone; but at the same time, I wonder what interest my brother-in-law has in my death—what benefit he is likely to derive from it. I also feel much surprised at his uttering filthy words, mixed with oaths and blasphemies. This was not his habit. He is extremely excited. He says, that since Satan has got his soul, he must likewise get mine.

On my side, the excitement becomes greater; I speak aloud to the crowd. The meaning of my speech being, that I fear nobody; that God is with me; that I am proud of having returned to better sentiments. I feel quite able to fight against Satan himself, because I am assured that I shall have an all-powerful assistance, already made manifest by the total absence of fatigue, fear, and want of food.

Whilst I am talking in this strain, my eyes fall on a damp place in the street or lane. The said place is much darker than the rest. (Water had probably been spilt there.) I fancy that it has the shape of a large hide. It is the devil's skin. I am told that my prayers and my faith have triumphed over Satan. I repeatedly trample on his remains, and only leave off to address the multitude around me. Fortunately my harangue is in French. They perhaps do not understand what I say; but they well enough perceive that I am not all right. A public-house is hard by, in which I hear music and songs. The airs are French. They are interrupted only by the voice of my brother-in-law, who exclaims that they must have my life, because he is sure I am not yet in a proper state for salvation. A young man comes out of the tavern (I perfectly recollect this incident), and offers me a glass of porter, which I decline to accept, because, said I, I have promised to my God henceforth to abstain from *fermented* drinks.

Some others among the crowd are not so kindly disposed in my favour. They would perhaps handle me somewhat rudely for my incomprehended discourse, were it not for the timely interference of a policeman, who has doubtless been enabled to perceive that if noisy I am not a dangerous character. In answer to his questions, I inform him that I am the sport of the infernal *puissance*, who want to get possession of my soul, and who have caused me to be hunted down in this city like a malefactor, a madman. The officer shows me much kindness. He endeavours to prove that I have nothing to fear; he sees that I am a stranger, and would the less on that account let me be insulted. I then say that I am homeless, without one single acquaintance in London, but with money enough to pay for a bed. The policeman asks me if I should have no objection to sleep in a poor-house. On my reply that I have none whatever to any place in which I may pass the night, he takes me to the station, to communicate with the chief officer about what is to be done with me. Here, too, I receive a good *accueil*; but the chief officer cannot take upon himself to send me to the poor-house; I must sleep in a lodging-house. I am, therefore, conducted by the policeman, who has brought me to a decent place, where I am recommended to the landlord. Before proceeding any further, I shall here state that several times in the streets, and especially whilst in the police-station, I most distinctly heard again a ringing of bells, as if coming down from above. The sound was sweet, harmonious, and seemed to be produced by silver bells. Another strange particular, the sky appeared to be illuminated by immense and innumerable round lamps, while there was now and then something like the noise created by the fall of hailstones.

I ask what o'clock it is. They inform me that it is nearly one. This is an eating-house, for many persons are at table, taking some food or a glass of beer. I should believe that they are carriers. I am told that the house keeps open all night, on account of the customers coming from the country. The room to which I am conducted is very spacious, and of neat appearance. It contains five beds, three of which are already occupied. I am scarcely in mine when I hear again from outside the voice of my brother-in-law more threatening than ever. He will not let me sleep. With Satan's assistance, he will get into the room; he will torment me to death. Then I fancy that he is in the yard, creating the same rattling noise as I heard once, by furiously driving an empty tumbrel round a circus. He stops now and then; but it is to laugh a sarcastic laughter, to call me hypocrite, to defy God, or to indulge in an obscene discourse. This lasts until after daybreak. I am still wide-awake, though I have not been in the least afraid; for there are two other voices close to my ears. They whisper to me that I have defensors, before whom Satan himself trembles. On my left I am addressed by my guardian angel, who informs me that I have been left to his care by our Lord. He says that I know him; that we were great friends; for he is the son of a neighbour of ours, with whom I used to play in my infancy. He died before he was ten years of age, more than twenty-five years ago, and became an angel in heaven. From that time he has been directed to watch over my actions. Had he been allowed to speak to me before, he would certainly have given me good counsels. For a number of years he has seen that I was running to eternal ruin, and he could do nothing but weep over my disorders, and pray that my eyes should be open. I have many friends in heaven, many relations who also interceded for my salvation. But *what was written was written.* I was destined to rush headlong to the very brink of destruction.

I then ask my guardian angel if he was not with me already, when I lay on a sick bed in Derry. He says he was; but he did not speak to me. I was then addressed by my full cousin, a young man of about twenty-seven years when he died, and who was a priest. To my question whether I shall be saved, my guardian angel gives no answer; but I hear, on my right, another voice, which says that I shall.

This voice is clearer and more distinct than the first. It is the voice of God Almighty himself, who deigns to communicate with me. I listen in awe and silence to the revelations that are being made. They generally relate to the destiny of my family and friends in the world to come. Parents, brothers, sisters, uncles, aunts, &c., all have the secret of their respective fates unfolded before me. Every life is minutely reviewed one after the other; every action, good or bad, carefully weighed. It is incredible how there is nothing forgotten or overlooked; it seems as if an every moment account-book had been kept, not only concerning the deeds but the thoughts and intentions of each. Most of them are doomed to suffer for ever; some for a certain length of time, and *one*, only *one*, is to obtain the kingdom of heaven. Then do I recollect a passage of the scripture, which I thought I had forgotten: *Multi enim vocati, pauci verò electi.*

It is a long time since the men have got up, I am still listening. Sometimes I presume to venture a question as to my future line of conduct. Every time I receive kind instructions for my guidance. Lastly, my imagination carries me to a scene hitherto unknown. I behold a sea of fire, into which an invisible hand precipitates the sinners, who have all preserved their human forms. As they appear one by one before the Supreme tribunal, I hear these redoubtable words from the Almighty: *Allez, fils de Satan, allez brûler dans le feu de l'enfer.* Although free from fear, I cannot help exclaiming more than once: "*O mon Dieu! que votre justice est terrible!*" I feel that I should like to sleep now; but I do not dare, for fear of displeasing God. The voice lets me know that I can rest myself after a short prayer. I therefore pray until I fall asleep. It must be at least five o'clock.

My sleep has been quite refreshing, not at all troubled by bad, terrifying dreams. It is breakfast-time when I get up. The voice on my right is gone; but my guardian angel is still here. He says he will not leave me. After dressing, I kneel down by the bed-side and say my morning prayers. My mind is much at ease. I have more confidence in myself; but no arguments could persuade me that the many events of last night are not real; everything must be true.

When I have done praying, I come down stairs. There are people engaged in breakfast. I ask for a cup of tea, with toast. They also bring me a little slice of ham, which I leave untouched, because I have no appetite to taste it. The rain has been falling a part of the night. It is not over yet. I wait in the room until it has abated. Then I resume my random strolls. I imagine that everybody knows what took place last night. Again the cries of *There is the madman* reach my ears. Whatever way I may go, they follow me; I cannot get rid of them. After several hours passed in moving about, like a mere machine, I find myself out of town, in the open fields, with only a few scattered houses in sight. Here I hope that I shall be more quiet. Although I was very thirsty, I had not dared to step into any place for refreshment, because I feared to be recognised as the madman of yesterday. My guardian angel, whose advice I ask for, tells me that I may take ginger-beer, but nothing else. The sky has cleared up; I sit down on the grass to rest myself a little. The place I have chosen is in the vicinity of a railroad. A train is coming, and, as it runs by, I distinctly and repeatedly hear the same annoying cry, *There is the madman*, as if all the passengers were acquainted with my history. I am extremely tired; I should like much to stop a little longer; but an invisible force bids me leave the spot and move on. I thus continue on my feet for some more hours, listening to the voice within me, and at times answering half aloud. I bend my steps back to town again, whither I am accompanied by the unceasing cry, to which I now submit with less reluctance. It must be late in the afternoon. The sky is overcast. I begin to be anxious about a place of rest. At last I find a chapel, and sit down at the door. I remain there for some time. The sudden idea strikes me that I am about to die; indeed, I feel something like two lobsters creeping up inside my chest. They are sucking my blood; and a voice tells me that I have but a few minutes more to live. This frightens me. My conscience is not in a right state yet: I am afraid to die. I go on in search of a chemist's shop, where I hope to obtain some relief. When I have found one, I complain of exhaustion, and ask for any strengthening medicine. The chemist gives me a cordial composed of—I don't know what,—which I swallow in the utmost confidence. I feel a little better, but not so well as to drive all fears of an imminent death away. My wishes are now to get to a Catholic chapel, and there to apply to a priest for confession. I therefore inquire about the nearest place of Catholic worship; I am directed to one about two miles off. Thither I direct my tottering steps: I find the door open, but no priest in. An old woman, whom I ask for him, says that she cannot tell me where he is. I leave this chapel to look for another: new wearisome stroll of nearly one hour. There is the object of my search, at last; but the entrance-door is locked: no possibility for me to get in. What to do? It is growing dark. The rain falls in large drops; I have no shelter, and I would not step into any public-house for fear of being at once recognised as the madman, and, as such, exposed to the abuse, perhaps to the blows, of the people. I come to the conviction that there shall be no rest for me until I have found out the inn in which I slept last night. I imagine that I shall be able to find it, and it is only after much time has been spent in walking at random that I perceive my presumptuous mistake. During all the time, the harassing cry of *There is the madman* has not ceased to sound by my ears. I again see and hear my persecutors beside me; now and then, too, the voice of my guardian angel keeps me up, as well as the silvery chime from above: this especially takes place when I have been praying fervently. Meanwhile, the rain has not abated; I am wet through; it is a late hour in the night, for I see lights nowhere except in very few public-houses. I have made repeated applications for a bed—all in vain. There was no accommodation. My resolution is now to pass the night out, and, as the rain prevents me from sitting down, to walk on until daylight. I reach a sheltered place, where, for want of a seat, I have been standing up for some time, when a policeman passes by. He asks me how it is that I am there at such a late hour. I tell him, that I could not find any lodgings, &c. . . . He can see by the gas-light that my clothes are very wet, and I appear to be extremely fatigued. He wishes to afford me a shelter for the remainder of the night at the police-station. I follow him; but the head officer cannot allow that I should stop in, because, says he, there is no charge against me. On the kind request of my guide, he, however, consents to send me to a workhouse, and writes a few words to that purpose, which he hands to the policeman. On our way to the poor-house, my imagination again works on my mind. I fancy that we are closely followed by an evil spirit, under the shape of a wolf, and with a human voice. I often complain to the officer that there is a demon behind us, who throws at me the same white-coloured liquid from which I formerly suffered so much. The dreaded shower burns all my body like boiling lead; it is accompanied with imprecations and fits of laughter from my pursuer. We arrive at the poor-house. They give me a bed, in which I soon fall asleep.

This first night has been quiet. When I awake in the morning, I expect that they are going to dismiss me; but I must wait for the doctor's visit. The medical gentleman easily perceives that I am not so well as I think. He cannot grant my discharge, unless I have a place where to go to. I feel quite surprised at the answer. I give way to despair, and reason leaves me altogether. The sight and hearing, so much impaired already, may now be termed mere organs of delusions. Besides mine, there are five beds in the room. In one of them I see a miserable victim, like myself. The four others are occupied by infernal spirits of the first order. They are the rebellious angels who presumed to revolt against God Almighty. Here, also, I shall meet with new attacks from my brother-in-law. I don't see him: I hear his voice and oaths as if he were in a room below. He said that it was himself who last night pursued me with the burning liquid, when on my way to the workhouse with the policeman. I shall not so easily escape now; for I am shut in, and he has powerful friends with him,—he means the evil spirits. He then discloses to me the secret and uncomprehended motives of his unceasing persecutions. I have done him no harm whatever; we ought, therefore, to be still on the same terms of good friendship as we were formerly. All this he cannot deny. However, he hates, he abhors me, and will only be happy when he sees me a corpse. My death must be the sinner's death. There must not be any time left for repentance; because, non-content with selling his own soul to Satan, he has likewise disposed of mine. The condition imposed by the Prince of Darkness is, that I shall die in my present state of sin. It appears that Satan sets a great value upon my soul. My brother-in-law informs me that 15,000 francs are the terms of the agreement in which I am, unknown to myself, so seriously concerned. I wonder much how my soul may be so eagerly sought for by the *Evil One*. My brother-in-law's soul fetched only 80*l*. Is mine any better? I then learn that God has decreed, in his inscrutable wisdom, that I shall obtain a place in the kingdom of heaven. Satan is aware of it. He also knows that, after a life of sins, I am destined to endure great sufferings, and to show sincere repentance before departing this life. He therefore gives here another instance of his well-known presumption; though he is obliged to confess that his own power cannot prevent the accomplishment of my *destiny*, he wants once more to try if he will be able to surprise the divine vigilance.

During the first days my fears of a sudden death are extreme. Twice or three times I escape from my bed, because I fancy that one or two of the boards of the floor are lifted up to give passage to my brother-in-law, whose face I don't see, but whose threats I hear. He will shoot me with a pistol. He has received from Satan the power of changing his natural form into a small animal, and to resume it as soon as he has got into my room. I also imagine that the melted lead is poured over my body from above my bed, through a small tube worked by Satan himself. I see him. He has taken the form of a black rat with red flaming eyes; he laughs at me, and says, he must have my soul. At night I behold frightful scenes; the men, whom I suppose to be evil spirits, assume horrid shapes; they are in perpetual motion, and all throw at me the burning liquid. The other patient to whom I have alluded as a victim has, like myself, to struggle against the same tormentor. He is possessed of extraordinary patience; I remark that he never swears, and if he does not pray, his frequent exclamations, such as "Lord have mercy upon me," show that he is a true believer. I become interested in his favour; I cannot help taking his defence (in words) whenever the *Infirmier* and another who styles himself *the Doctor* beat him in order to reduce him to silence.

(I have since recognised that patient in Andrews, who is now in this establishment.)

I have no rest: a voice tells me that prayer alone shall bring relief. I therefore pray for hours, for days and nights without interruption, except when I cannot go on from exhaustion. I wont take any food. Everything is loathsome to me, and besides, the food which is presented to me is the usual nourishment of the devils; it would be poison for me. Now and then I drink a drop of water, but every time after praying that it should be changed into a wholesome beverage. The conduct of my co-patients is not calculated either to alter my opinion in their respect. One of them especially has nothing but oaths or filthy words in his mouth. The *infirmier* himself is not better. Whenever I make a noise he abuses me in a low language, and even strikes me with his fist. Their imprecations and ill-treatments, far from compelling me to silence, only tend to redouble my excitement. Unlike to Andrews, I often upbraid them for their rudeness; I say that I don't fear them. They are demons, I know; they may kill me; they shall have my body; but my soul, never. I am resolved to suffer and to forgive. I exhort them to repentance by repeatedly saying, *Repentez-vous, repentez-vous; car le royaume des cieux est proche*, &c.

My prayers and exhortations, being expressed aloud and in French, produce on my hearers no other effect than that of irritating them the more; for sleep has become quite impossible. From the beginning, I have been tied up, head, hands, and feet, in my bed. One would think that all movements are impeded. I however keep in constant fidgeting; it is truly surprising how I may still have so much strength. Every morning I am untied that I may wash myself; but as soon as the process of washing is over, I am generally bound again, with the difference that my head and hands are left free. At times, myriads of white flies are sent into my bed; they stick to my skin like leeches; they suck up my blood, and their stings create considerable pains. Every night the room is changed into a kind of infernal laboratory. There is Satan writing cabalistic characters on the wall; there is one of his *suppôts* standing by my side, and keeping under my nose a sulphuric match which he forces me to inhale. This unearthly being's stature is gigantic. There are other demons in various shapes moving or crawling about. I see toads and frogs of enormous size. They torment the patient Andrews. There are black pigs intended for devouring my feet, as they were once by a dog. I expect new tortures, but in a spirit of resignation; the break of day generally causes my visions to disappear. I then fall into a kind of sleep of very short duration. As soon as I am awakened, I commence praying again, and only leaving off doing so when I am too much exhausted. For many days and nights I have thus prayed for—1st, my family and friends; 2nd, for mere acquaintances; 3rd, for even those whom I suppose to be my enemies. My mind is not only occupied in praying;

God again unfolds to me the destinies of the many persons with whom I am acquainted. I am very inquisitive; I wish to know the fate of the most celebrated personages about whom I have read in history, and especially of the Frenchman who acted the most prominent parts in our first revolution. I learn that all the kings of France have become the subjects of Satan, except a single one, Louis XVI. As to the Terrorists, they are in hell, along with our greatest writers, such as Voltaire, J. J. Rousseau, &c. Many, many persons whom I expect to be saved, are not; and a few are whom I have looked on as *Jesuits.* If in my prayers, any worldly thought crosses my mind, I immediately hear the voice say, *Encore des idées d'orgueil,* or else, *Encore de l'envie ; encore l'amour des richesses.* All this must be made away with before I can enter the kingdom of heaven. Sometimes I am commanded to say a prayer which I have forgotten. Then my cousin prays for me. Almost every night I see a window where I know that there is none. There I behold Almighty God, and our Lord Jesus Christ, such as they are represented in Catholic pictures. Christ intercedes for me ; I distinctly notice a tear falling down his cheek, as if he were weeping on my sins. There also come two children, whom I take for the Infant Jesus and John the Baptist. Their mothers are with them ; they all want to implore God's forgiveness in my favour. The sky outside is now bright, now it assumes a lurid appearance, according as my prayers come from a contrite or a doubtful heart. Towards the latter end of my confinement in the house I am informed that I shall be admitted into heaven. My trials are over ; I need not fear or doubt any longer. I am to be taken to the celestial palaces in God's own chariot. I feel very happy. Shortly after, the voice tells me that my soul is gone. My body is now only animated by a *souffle.* I cannot well understand, but I believe. Now, too, I fancy that God reveals to me the future destinies of the world. The kingdom of Heaven is at hand : mankind shall perish within a few days by a general conflagration. The plague is raging in London and many cities on the Continent. In France the demon of murder and suicide exercises his sway over the whole population. Last revolution in Paris ; the soldiers are fighting against the people, then against each other, until there is but one man surviving, who shoots himself. Many times I imagine that I hear a sinister voice in London : it says, "*Visited such, such, and such streets; all dead:* may God forgive us !" &c.

Such were, Monsieur le Docteur, the strange thoughts by which my mind was engrossed when I was removed from the poorhouse. The gentlemen who came for me did not surprise me in the least on announcing that I must get up, for they were to take me away. I firmly believed that I was dead, and likely about to be admitted into Heaven. Nothing, in my imagination, could be expected. On our way hither, I saw houses, trees, carriages, passengers, all as it is on earth; but I would have been averse to the idea that they did not belong to another world, a kind of medium between earth and heaven.

When we alighted here, I came to think that I was to be shut in for a limited space of time. This was the last expiation for my sinful life. I kept in *sullen* silence, because it was my belief that *mutism* was the condition, *sine quâ non*, for my speedy ascent to heaven. The attendants and patients with whom I was placed, I considered as new temptators, whose attacks I should have to resist. Thus I fancied that my duty was to walk up and down the gallery with the least possible rest, and taking care always to tread on the same boards. I also considered it my duty to obey the attendants whenever they said, *I will.* In the yard the trial was of another kind. "I must not," said I, "let any one make his way on the same path as I do; I must drive him away by constantly walking around him, and surrounding him with invisible lines, as the spider weaves his nets around flies."

Once, I recollect, they retired to the shed. I took up my post right against them, and stood up for a long time there, moving three steps backward and forward. It seemed to me that I was ordered to do so some hundred times before allowing myself any rest. On the three or four first nights, you may remember, Monsieur le Docteur, that I was in an excited state. Indeed, you were so kind as to lend me a French book, which I did not dare to peruse, for fear it should be a snare set against my soul. In my room I used to pray and speak aloud, as I had done in the pauper-house, for I felt convinced this establishment (the purgatory) was swarming with invisible beings, some in need of my prayers, others of my exhortations. Any person coming at that time to invite me to be quiet, was sure to be taken for a temptator, at whom I threw the malediction, *Vade retrò, Satanas.* Fortunately, I soon was enabled to see things in their proper light. But to what causes shall I ascribe my quick recovery, if not to God's mercy first, and then to you, Monsieur le Docteur, to the Rev. Mr. Murray, and to the attendants of your choice. Had I been so roughly treated here as I was in the poor-house, my firm belief is that I should never have recovered. Your constant kind attentions to me, your willingness to grant me whatever I may desire, such as books, newspapers, extra diet,—these are titles to my gratitude, which I shall never forget. In my present helplessness, I can only say what many others will repeat after me: May you, Monsieur le Docteur, and your family, enjoy the happiness which you deserve so well!

I forgot, Monsieur le Docteur, to mention to you the fact that, during my stay in the poor-house, the state of my bowels was always that of costiveness, and that my water was of a red colour. Now I feel as well as I can possibly be, were it not for the itchings which I still feel now and then, and which disturb my sleep to some degree. I suppose that they will disappear in time.

I have given you many tedious details; you will find many inconsistencies, perhaps, in the course of my narrative; but you know that I had only to write down the ideas of a delirious brain. There can be no *logique* expected from such a source. My only endeavour has been to relate the truth, and nothing but the truth, a condition which my vivid recollections made quite easy. I sincerely wish, Monsieur le Docteur, that it should meet your approval.

L. D.

---

